# Medium-Term Exposure to Traffic-Related Air Pollution and Markers of Inflammation and Endothelial Function

**DOI:** 10.1289/ehp.1002560

**Published:** 2011-02-24

**Authors:** Stacey E. Alexeeff, Brent A. Coull, Alexandros Gryparis, Helen Suh, David Sparrow, Pantel S. Vokonas, Joel Schwartz

**Affiliations:** 1 Department of Environmental Health and; 2 Department of Biostatistics, Harvard School of Public Health, Boston, Massachusetts, USA; 3 VA Normative Aging Study, VA Boston Healthcare System, Department of Medicine, Boston University School of Medicine, Boston, Massachusetts, USA

**Keywords:** adhesion molecules, air, cardiovascular, environmental, outdoor air, roadway proximity

## Abstract

**Background:**

Exposure to traffic-related air pollution (TRAP) contributes to increased cardiovascular risk. Land-use regression models can improve exposure assessment for TRAP.

**Objectives:**

We examined the association between medium-term concentrations of black carbon (BC) estimated by land-use regression and levels of soluble intercellular adhesion molecule-1 (sICAM-1) and soluble vascular cell adhesion molecule-1 (sVCAM-1), both markers of inflammatory and endothelial response.

**Methods:**

We studied 642 elderly men participating in the Veterans Administration (VA) Normative Aging Study with repeated measurements of sICAM-1 and sVCAM-1 during 1999–2008. Daily estimates of BC exposure at each geocoded participant address were derived using a validated spatiotemporal model and averaged to form 4-, 8-, and 12-week exposures. We used linear mixed models to estimate associations, controlling for confounders. We examined effect modification by statin use, obesity, and diabetes.

**Results:**

We found statistically significant positive associations between BC and sICAM-1 for averages of 4, 8, and 12 weeks. An interquartile-range increase in 8-week BC exposure (0.30 μg/m^3^) was associated with a 1.58% increase in sICAM-1 (95% confidence interval, 0.18–3.00%). Overall associations between sVCAM-1 and BC exposures were suggestive but not statistically significant. We found a significant interaction with diabetes—where diabetics were more susceptible to the effect of BC—for both sICAM-1 and sVCAM-1. We also observed an interaction with statin use, which was statistically significant for sVCAM-1 and suggestive for sICAM-1. We found no evidence of an interaction with obesity.

**Conclusion:**

Our results suggest that medium-term exposure to TRAP may induce an increased inflammatory/endothelial response, especially among diabetics and those not using statins.

There is strong epidemiological evidence that short-term air pollution exposure (i.e., < 24 hr to 3 weeks) is related to mortality and other cardiovascular events (Brook et al. 2010). Much of this evidence involves exposure to ambient particulate matter (PM) with aerodynamic diameter ≤ 2.5 μm (PM_2.5_), which comprises many components and varies regionally. Contributions of specific components and sources to these effects are not well understood but are critical for informing the development of regulations.

Short-term exposure studies often use stationary monitors to estimate exposure in a nearby region. However, specific components of traffic-related air pollution (TRAP) vary substantially within cities, and traffic variables may contribute to this variation (Brauer et al. 2003; Clougherty et al. 2008; Kinney et al. 2000). This suggests that a geographically based approach could substantially improve assessment of black carbon (BC) exposure.

Long-term exposure to TRAP (i.e., averages ≥ 1 year) has also been associated with cardiovascular mortality, often based on studies using nitrogen dioxide as a surrogate for traffic pollutants and spatial modeling of exposures (Brunekreef et al. 2009; Gehring et al. 2006; Yorifuji et al. 2010). In a Boston, Massachusetts–area case-crossover analysis, we reported an association between mortality and BC exposure on the day before death, using spatiotemporally modeled BC exposure estimates as a marker of TRAP (Maynard et al. 2007).

Intercellular adhesion molecule-1 (ICAM-1) and vascular cell adhesion molecule-1 (VCAM-1) are markers of inflammation and endothelial function that are expressed on cell surfaces and are also found in soluble form in the plasma (sICAM-1 and sVCAM-1). These markers are independently and jointly associated with increased risk of cardiovascular disease (Albert and Ridker 1999; Pradhan et al. 2002; Rana et al. 2011; Ridker et al. 2003). Recent studies have reported associations between inflammatory markers and short-term exposure to TRAP, but studies have varied in both PM exposure type and inflammatory markers examined (Delfino et al. 2008; Madrigano et al. 2010; Zeka et al. 2006).

Obesity and diabetes are becoming increasingly prevalent, and these conditions may increase susceptibility to the adverse health effects of air pollution (Forastiere et al. 2008; O’Neill et al. 2005; Zanobetti and Schwartz 2002). Prospective and randomized clinical trials, as well as laboratory studies, have demonstrated that statins, a widely prescribed class of drugs with anti-inflammatory and antioxidant activity (Haendeler et al. 2004), can decrease C-reactive protein (CRP), ICAM-1, and VCAM-1 levels (Albert et al. 2001; Blanco-Colio et al. 2007; Liang et al. 2008; Montecucco et al. 2009). Thus, it is plausible that statin use could attenuate proinflammatory effects of air pollution, and reduced effects of air pollution on inflammation and heart-rate variability among statin users compared with nonusers have been reported (Schwartz et al. 2005; Zeka et al. 2006).

The primary objective of the present study was to estimate the cumulative effects of exposure to TRAP over several weeks on inflammation and endothelial function. Previous findings based on BC measured at a central-site monitor suggested associations between inflammatory markers and air pollution exposure averaged over the prior 4 weeks (Zeka et al. 2006). Because BC concentration varies spatially and averaging over a longer period of time decreases the temporal variation in the data, we wanted to examine effects of 4- to 12-week average exposures estimated using a land-use regression model. We chose sICAM-1 and sVCAM-1 as outcomes because they reflect changes in inflammation, are associated with cardiovascular disease risk, and are relatively stable within individuals over 4-week periods (Eschen et al. 2008).

We hypothesized that increases in medium-term BC concentrations estimated by land-use regressions would be associated with an increased inflammatory response in elderly men in the Greater Boston area. We also examined whether that effect was modified by statin use, obesity, and diabetes.

## Materials and Methods

### Study population

We studied participants in the Normative Aging Study (NAS), a longitudinal study established by the VA in 1963 (Bell et al. 1972). In brief, the NAS enrolled 2,280 men from the Greater Boston area who were initially free of known chronic medical conditions. All participants provided written informed consent, and the study was approved by the institutional review boards of all participating institutions. Participants visited the Boston VA Hospital study center every 3 years to undergo physical examinations. At each of these visits, blood samples and extensive physical examination, laboratory, anthropometric, and questionnaire data were collected. Information about cigarette smoking, medical history, and medication use were obtained by self-administered questionnaire. Each subject was interviewed to confirm the identity and purpose of medications used, and all new disease diagnoses were noted.

Diabetes was defined as a physician diagnosis of diabetes, and obesity was defined as a body mass index (BMI) of at least 30 kg/m^2^. Self-reported data on diabetes status and statin use were updated at each study visit. In addition, BMI and obesity were updated based on height and weight measurements at each visit. Thus, NAS data reflect changes in disease status and medication use over time.

Measurements of sICAM-1 and sVCAM-1 began in 1999. For the present study, we included the 642 NAS participants with at least one measurement of sICAM-1 and sVCAM-1 and whose home address was in the Greater Boston area (1,423 total person-visits). Subjects who moved out of the area were excluded.

### Measurements of sICAM-1 and sVCAM-1

Blood samples routinely collected during medical exam visits from 1999 through 2008 were analyzed for sICAM-1 and sVCAM-1 in N. Rafai’s laboratory at Children’s Hospital Boston (Boston, MA). Plasma sICAM-1 and sVCAM-1 concentrations were measured in duplicate using the enzyme-linked immunosorbent assay (ELISA) method (R&D Systems, Minneapolis, MN), with a sensitivity of 0.35 ng/mL for sICAM-1 and 2.0 ng/mL for sVCAM-1 (Lim et al. 1999).

### BC exposure prediction

The BC exposure model and the stationary air monitors used to develop the model have been described in detail previously (Gryparis et al. 2007). Briefly, 82 sites were used; most sites measured BC continuously using aethalometers, and other sites collected particles on a filter over 24 hr and measured elemental carbon (EC) using reflectance analysis. The monitoring data used to develop our model included 6,031 observations from 2,079 unique exposure days.

Using a spatiotemporal model that we developed and validated previously (Gryparis et al. 2007), we estimated the 24-hr average BC concentration at each geocoded participant address. Predicted daily concentrations showed a > 3-fold range of variation in exposure across measurement sites (adjusted *R*^2^ = 0.83). A validation sample at 30 additional monitoring sites showed an average correlation of 0.59 between predicted and observed daily BC levels. We averaged the 24-hr predictions to form estimates for the 4, 8, and 12 weeks before each participant visit. We also averaged the 24-hr predictions to form estimates for the 4-week average during the 5–8 weeks before the study visit and the 4-week average during the 9–12 weeks before the study visit, which are components of the 8- and 12-week averages, to use as a sensitivity analysis.

Covariates in the BC prediction model included measures of land use for each address (cumulative traffic density within 100 m, population density, distance to nearest major roadway, and percent urbanization), geographic information system (GIS) location (latitude, longitude), daily meteorological factors (apparent temperature, wind speed, and height of the planetary boundary layer), and other characteristics (day of week, day of season). The Boston central-site monitor was also included as a predictor to reflect average pollutant concentrations over the entire region on each day.

Separate models were fit for warm and cold seasons. Interaction terms between the temporal meteorological predictors and source-based geographic variables allowed for space–time interactions. Regression splines allowed main effect terms to nonlinearly predict exposure levels, and thin-plate splines modeled the residual spatial variability not explained by the spatial predictors. A latent variable framework was used to integrate BC and EC exposure data, where BC and EC measurements were treated as surrogates of some true, unobservable traffic exposure variable; see Gryparis et al. (2007) for further details.

### Estimation of health effects

We log-transformed sICAM-1 and sVCAM-1 levels to increase normality and stabilize variance in the residuals. Model covariates were selected *a priori*, and all models included age, BMI, diabetes, smoking status, pack-years, and season. We used mixed models to account for correlation among measurements on the same subject across different medical visits. Mixed models have the form


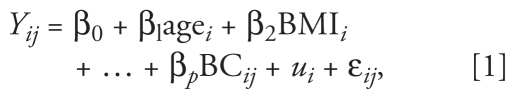


where *Y_ij_* is log(sICAM-1) or log(sVCAM-1) in subject *i* on day *j*, and *u_i_* represents a subject-specific intercept that reflects unexplained heterogeneity in the outcome. BC averages and model covariates are modeled as fixed linear effects, and *u_i_* is modeled as a random effect. We assume that the *u_i_* are generated from a normal distribution with common variance, yielding the simple compound symmetry variance structure. This model requires estimation of two variance components, which represent between- and within-subject variation. Models with unbalanced data (i.e., varying numbers of repeated measurements on each subject) typically yield accurate estimates of within-subject variation, provided a sufficient number of repeated measurements contribute to the estimate.

Models used to examine effect modification by obesity, diabetes, and statin use included interaction terms that allowed associations between BC and the outcomes to vary among subgroups. Diabetes, obesity, and statin use were treated as time-varying covariates, where the status was updated at each visit to reflect changes since the last visit. The percentages of subjects whose status changed for these factors over the study period were 3% for diabetes, 9% for obesity, and 21% for statin use.

We also performed a sensitivity analysis to investigate whether the interaction with statin use was heavily influenced by the people who began taking statins after the study period began. Specifically, we limited the interaction analysis to the participants who never used statins during the study period (*n* = 284) and those who used statins throughout the entire study period (*n* = 223).

Because the outcomes were log-transformed, effect estimates are reported as percent changes in sICAM-1 and sVCAM-1 concentrations associated with a 0.30-μg/m^3^ increase in BC, which corresponds to the interquartile range (IQR) for average BC exposures over all three time intervals (4, 8, and 12 weeks). A level of α = 0.05 was used to determine statistical significance.

## Results

### Descriptive data

**Figure 1:**
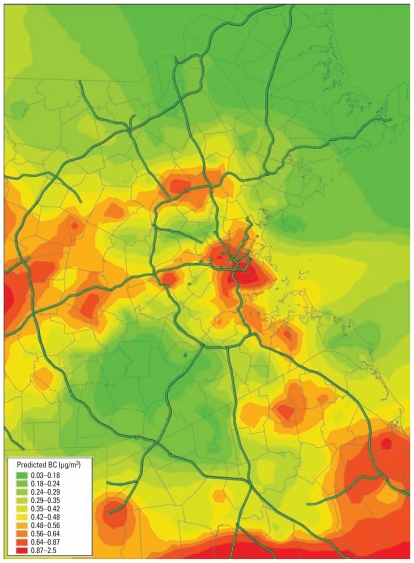
Predicted average BC concentration in the Greater Boston area over a 4-week period.

Subjects were elderly, with a mean age of 72 years (range, 56–100 years) at the first study visit (Table 1). Most subjects were overweight (median BMI, 27.5 kg/m^2^; range, 18–52 kg/m^2^). Average 4-week BC exposure estimates ranged from 0 to 3.26 μg/m^3^ (mean ± SD, 0.42 ± 0.30 μg/m^3^; median, 0.37 μg/m^3^; IQR, 0.30 μg/m^3^). For both 8- and 12-week exposures, exposure estimates were 0.42 ± 0.29 μg/m^3^ (median, 0.38 μg/m^3^; IQR, 0.30 μg/m^3^). Figure 1 illustrates the spatial distribution of predicted BC in the study area during an arbitrary 4-week period (February 2001). The distributions of BC exposures looked similar among subjects classified according to obesity, diabetes, statin use, or smoking status, and we found no significant differences in the mean BC exposure assessed at baseline.

### Regression analysis

We found significant positive associations between sICAM-1 and BC averaged over the previous 4, 8, and 12 weeks (Table 2). Associations between average BC for 4, 8, and 12 weeks and sVCAM-1 were in the same direction, with slightly smaller effect size, but were not statistically significant. As a sensitivity analysis, we also estimated the effects of BC exposure in weeks 5–8 and weeks 9–12 separately. For sICAM-1, the association with the longer BC lags were positive with effect size slightly smaller than the effect size for weeks 1–4 and not quite statistically significant. A 0.30-μg/m^3^ increase in BC exposure during weeks 5–8 was associated with a 1.22% increase in sICAM-1 [95% confidence interval (CI), −0.10% to 3.00%], and during weeks 9–12, with a 1.09% increase in sICAM-1 (95% CI, −0.18% to 2.38%). For sVCAM-1, the estimated effects for the longer lags had the same effect size and direction as the effects for weeks 1–4 but were not statistically significant (data not shown). Overall, we saw the same pattern of effects in the lagged 4-week intervals, with only slight weakening in effect when the lags were considered individually rather than cumulatively.

### Regression analysis and interactions

**Figure 2:**
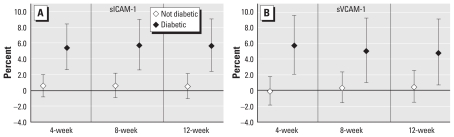
Estimated percent increase in sICAM-1 (*A*) and sVCAM-1 (*B*) levels by diabetic status, per 0.3-μg/m^3^ increase in BC, for 4-, 8-, and 12-week averages.

**Figure 3:**
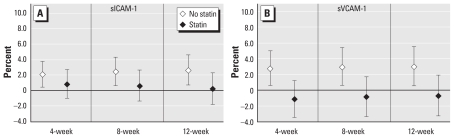
Estimated percent increase in sICAM-1 (*A*) and sVCAM-1 (*B*) levels by statin use, per 0.3-μg/m^3^ increase in BC, for 4-, 8-, and 12-week averages.

When we examined the effect of BC on sICAM-1 and sVCAM-1 by diabetes status, we saw effects only among diabetics, whereas nondiabetics showed no effect (Figure 2; interaction *p*-values < 0.05 for all models except 12-week BC and sVCAM-1). When we examined the effects of BC by statin use, interaction models suggested that effects were present only in participants who did not use statins, with little evidence of associations among statin users (Figure 3; interaction *p*-values < 0.05 for sVCAM-1 only). In contrast, we found no difference in the estimated effect of BC by obesity status on either outcome (data not shown).

We restricted our sensitivity analysis of statin use interaction to participants who never used statins or always used statins during the study period. The results were comparable, with the effect sizes estimated to be slightly larger in all categories in the restricted model, and statistically significant (data not shown). These differences could reflect some overall difference in health between statin users and nonusers because statin use was not randomized.

## Discussion

We found that address-specific BC exposures averaged over 4, 8, and 12 weeks were positively associated with markers of inflammation and endothelial dysfunction in this elderly cohort. Exposure estimates based on our validated land-use regression model are more accurate than estimates based on ambient monitoring, which are often used for cohorts of this size. Our analyses suggest that diabetics are more susceptible to adverse effects of TRAP than are nondiabetics, but we found no evidence of effect modification by obesity. In addition, we observed a null effect among participants who used statins and a positive association for those not taking statins, but further investigation is needed to clarify this potential effect.

### TRAP and inflammatory response

Several studies have examined the effects of short-term (i.e., < 24 hr to 3 weeks) TRAP and various markers of inflammation. For example, in a large epidemiological study, daily increases in ambient PM levels were positively associated with plasma fibrinogen levels, with the strongest association observed in participants with chronic obstructive lung disease (Schwartz 2001). In a study of a small (*n* = 29) elderly population, Delfino et al. (2008) examined inflammatory markers and PM components measured at each subject’s home for 1–9 days on average. The authors reported that several traffic-related components were significantly associated with CRP and interleukin-6, and observed positive but nonsignificant associations with sICAM-1 and sVCAM-1.

In other studies of NAS participants, traffic-related PM (BC and organic carbon) exposure has also been positively associated with plasma total homocysteine concentrations (Park et al. 2008), and this association is modified by polymorphisms in genes related to oxidative stress (Ren et al. 2010). PM_2.5_ and BC averages of 1–3 days measured at the central site have been associated with increased vascular cell adhesion in NAS participants (Madrigano et al. 2010). Traffic-related PM (particle number and BC) has also been positively associated with inflammatory markers (CRP, white blood cell count, sediment rate, and fibrinogen) in NAS participants, with stronger associations with particle numbers than with BC and stronger associations with BC averaged over 4 weeks than averaged over 48 hr or 1 week (Zeka et al. 2006).

Few controlled studies of PM on human inflammatory response have been conducted, but one reported that plasma fibrinogen increased after exposure to urban PM (Ghio et al. 2000), and another reported that peripheral neutrophils, sVCAM-1, and sICAM-1 increased after a 1-hr exposure to diesel PM (Salvi et al. 1999).

### Mechanisms and interactions

Diabetics have impaired endothelial function compared with nondiabetics (Calles-Escandon and Cipolla 2001), and there is increasing evidence that diabetics are more susceptible to the effects of air pollution. Inverse associations between 60-day average BC exposure and brachial artery flow-mediated dilation (FMD) were stronger in diabetics than in nondiabetics (O’Neill et al. 2005). Associations between 24-hr exposure to PM_2.5_ and FMD were stronger among diabetics with markers of severe insulin resistance compared with other diabetics (Schneider et al. 2008).

In contrast with our findings, obese NAS participants have been reported to have stronger associations between short-term BC exposure and plasma CRP, erythrocyte sediment rate (Zeka et al. 2006), and sVCAM-1 (Madrigano et al. 2010) than nonobese participants.

Our finding that statin users are less susceptible than those not taking statins is generally consistent with other studies. In diabetics, stronger associations of PM_2.5_ and BC with sVCAM-1 were reported among those not using statins than in statin users (O’Neill et al. 2007). Associations between CRP and traffic PM exposures of 5 and 9 days were reported to be stronger among those using statins than those not using statins (Delfino et al. 2008). Statins promote endothelial nitric oxide release, which reduces cell adhesion, thus suggesting a mechanism for this association. However, we cannot rule out the possibility that statin use is an indicator of health status or some other unmeasured factor that may explain why BC did not appear to influence sICAM-1 and sVCAM-1 in statin users.

The question of which CAM is most closely associated with PM remains unresolved. Differences in the associations reported in epidemiological studies of the two CAMs could reflect differences in cell types that express the molecules or differences in the process of cleavage and shedding from endothelial cells. In the present study, the effects on both sICAM-1 and sVCAM-1 were consistent: The effect sizes were similar and the direction of the effects and the interactions were the same, even though we found differences in statistical significance. Thus, our study does not support the idea of a different underlying mechanism for the effects of TRAP on these two CAMs.

The expression of both ICAM-1 and VCAM-1 on the surface of vascular endothelial cells is associated with the formation of early atherosclerotic lesions. Although the relationship between the degree of cellular ICAM-1 and VCAM-1 expression and plasma concentrations of soluble forms is not entirely clear, multiple studies have shown that sICAM-1 and sVCAM-1 predict risk of cardiovascular disease (Albert and Ridker 1999; Pradhan et al. 2002; Rana et al. 2011; Ridker et al. 2003). In a recent study investigating the variability of sICAM-1 and sVCAM-1 measures over a 4-week period, Eschen et al. (2008) reported an estimated intrasubject variability of 7.6% for sICAM-1 and 9.5% for sVCAM-1, which suggests that these markers are relatively stable over a 4-week period.

### Exposure estimation

A major advantage of the present study is the use of a validated land-use regression model to characterize the individual-level differences in exposures instead of classifying exposure based on measurements at the nearest monitor, land-use regression models without BC measurements, or a weighted form of distance to roadway. Although the study is still limited by the lack of individual-level monitoring data at the home, our validation study suggests that our estimates are highly correlated with actual exposure measurements at locations in Boston other than those used to fit the model and are much more closely correlated with these measurements than are exposure estimates based on a central-site monitor. Although some exposure misclassification will still occur based on our model estimates, we expect that most of the residual error is Berkson type, based on a previous validation study for this model analyzing measurement error (Gryparis et al. 2009). Thus, we expect that the exposure misclassification will not bias effect estimates.

The lack of individual activity data also presents a limitation, but the NAS population consists of elderly men who spend a considerable amount of time at or near their home address compared with other population groups.

Our sensitivity analysis of the block lags of weeks 5–8 and weeks 9–12 shows slight attenuation compared with the cumulative lags, suggesting that the cumulative effect may be dominated by the more recent weeks. The effect sizes for cumulative exposures do not attenuate when averaged up to 12 weeks. The 4-week block lags do not isolate the effects of those time windows because each week is correlated in both space and time; thus, future studies to model the specific contributions of different lag weeks would be beneficial in understanding these effects.

### Sources and components

Many studies have examined PM at different diameters, but fewer studies have looked at which components of PM are associated with adverse health effects. Our study links a health biomarker with BC, a specific component of PM_2.5_. In addition, we consider BC to be a surrogate for primary traffic PM (a specific source of air pollution), where the spatial variability of BC on a given day reflects the variability of traffic in the region, which is the basis of the BC model we developed and used for this study.

We did not examine other pollutants such as total PM_2.5_ or PM_2.5_ components other than BC in this study. We think it is unlikely that total PM_2.5_ or any other copollutant is driving the effect we observed for BC because the BC model is based largely on the daily spatial variation exhibited by BC. Other components of PM_2.5_, such as sulfates and organic PM, are more homogeneous over the study region. Although we cannot completely rule out confounding by copollutants or other factors, the correlation between the model-predicted 4-week BC and the corresponding 4-week averages of PM_2.5_ measured at the central site was low (0.108), which supports our belief that the effects observed are not driven by any temporal correlation with PM_2.5_ or one of its components.

### Generalizability

A limitation of this study is the restricted demographics of the study population. Study subjects were all elderly men, most of them white. Thus, we cannot generalize our results to other populations. However, the elderly represent a particularly susceptible population, and the growth in the number and proportion of older adults in the United States is unprecedented: by 2030, the proportion of the U.S. population ≥ 65 years of age will double to about 71 million older adults, or one in every five Americans (U.S. Census 2005).

## Conclusions

We observed positive associations between BC exposures and blood levels of sICAM-1 and sVCAM-1, with statistically significant effect estimates for sICAM-1 in the population as a whole and for sVCAM-1 among diabetics and participants who were not using statins. Effects of BC on both markers appeared to be limited to diabetics and possibly those not using statins. Overall, our results suggest that exposure to traffic PM over 4–12 weeks may induce an increased inflammatory and endothelial response, particularly among diabetics, and that statin use may be protective against this effect.

## Figures and Tables

**Table 1:** Characteristics of the NAS population at first visit.

Covariate	Mean ± SD or *n* (%)
Age (years)	72.4 ± 7.0
BMI (kg/m^2^)	28.3 ± 4.2
Diabetes	126 (19.6)
Obesity	180 (28.0)
Statin use	242 (37.7)
Smoking status	
Former smoker	657 (69.7)
Current smoker	28 (2.9)
Never smoker	258 (27.4)
Pack-years^a^	30.4 (27.9)
sICAM-1 (ng/mL)	295.4 ± 80.6
sVCAM-1 (ng/mL)	1058.9 ± 351.8

a For current and former smokers.

**Table 2:** Estimated percent increase in sICAM-1 and sVCAM-1 levels per 0.3-μg/m^3^ increase in BC, for 4-, 8-, and 12-week averages.

	sICAM-1		sVCAM-1	
Time period	Percent increase	95% CI	Percent increase	95% CI
4 weeks	1.50	0.22 to 2.80*	1.00	−0.65 to 2.67
8 weeks	1.58	0.18 to 3.00*	1.20	−0.58 to 3.02
12 weeks	1.49	0.04 to 2.95*	1.26	−0.58 to 3.14

* *p* < 0.05.

## References

[b1-ehp-119-481] Albert MA, Danielson E, Rifai N, Ridker PM (2001). Effect of statin therapy on C-reactive protein levels: the pravastatin inflammation/CRP evaluation (PRINCE): a randomized trial and cohort study. JAMA.

[b2-ehp-119-481] Albert MA, Ridker PM (1999). The role of C-reactive protein in cardiovascular disease risk. Curr Cardiol Rep.

[b3-ehp-119-481] Bell B, Rose C, Damon A (1972). The Normative Aging Study: an interdisciplinary and longitudinal study of health and aging. Aging Hum Dev.

[b4-ehp-119-481] Blanco-Colio LM, Martin-Ventura JL, de Teresa E, Farsang C, Gaw A, Gensini G (2007). Elevated ICAM-1 and MCP-1 plasma levels in subjects at high cardiovascular risk are diminished by atorvastatin treatment. Atorvastatin on Inflammatory Markers study: a substudy of Achieve Cholesterol Targets Fast with Atorvastatin Stratified Titration. Am Heart J.

[b5-ehp-119-481] Brauer M, Hoek G, van Vliet P, Meliefste K, Fischer P, Gehring U (2003). Estimating long-term average particulate air pollution concentrations: application of traffic indicators and geographic information systems. Epidemiology.

[b6-ehp-119-481] Brook RD, Rajagopalan S, Pope CA, Brook JR, Bhatnagar A, Diez-Roux AV (2010). Particulate matter air pollution and cardiovascular disease: an update to the scientific statement from the American Heart Association. Circulation.

[b7-ehp-119-481] Brunekreef B, Beelen R, Hoek G, Schouten L, Bausch-Goldbohm S, Fischer P (2009). Effects of long-term exposure to traffic-related air pollution on respiratory and cardiovascular mortality in the Netherlands: the NLCS-AIR study. Res Rep Health Eff Inst.

[b8-ehp-119-481] Calles-Escandon J, Cipolla M (2001). Diabetes and endothelial dysfunction: a clinical perspective. Endocr Rev.

[b9-ehp-119-481] Clougherty JE, Wright RJ, Baxter LK, Levy JI (2008). Land use regression modeling of intra-urban residential variability in multiple traffic-related air pollutants. Environ Health.

[b10-ehp-119-481] Delfino RJ, Staimer N, Tjoa T, Polidori A, Arhami M, Gillen DL (2008). Circulating biomarkers of inflammation, antioxidant activity, and platelet activation are associated with primary combustion aerosols in subjects with coronary artery disease. Environ Health Perspect.

[b11-ehp-119-481] Eschen O, Christensen JH, Dethlefsen C, Schmidt EB (2008). Cellular adhesion molecules in healthy subjects: short term variations and relations to flow mediated dilation. Biomark Insights.

[b12-ehp-119-481] Forastiere F, Stafoggia M, Berti G, Bisanti L, Cernigliaro A, Chiusolo M (2008). Particulate matter and daily mortality: a case-crossover analysis of individual effect modifiers. Epidemiology.

[b13-ehp-119-481] Gehring U, Heinrich J, Kramer U, Grote V, Hochadel M, Sugiri D (2006). Long-term exposure to ambient air pollution and cardiopulmonary mortality in women. Epidemiology.

[b14-ehp-119-481] Ghio AJ, Kim C, Devlin RB (2000). Concentrated ambient air particles induce mild pulmonary inflammation in healthy human volunteers. Am J Resp Crit Care Med.

[b15-ehp-119-481] Gryparis A, Coull BA, Schwartz J, Suh HH (2007). Semiparametric latent variable regression models for spatiotemporal modeling of mobile source particles in the Greater Boston area. Appl Stat.

[b16-ehp-119-481] Gryparis A, Paciorek CJ, Zeka A, Schwartz J, Coull BA (2009). Measurement error caused by spatial misalignment in environmental epidemiology. Biostatistics.

[b17-ehp-119-481] Haendeler J, Hoffmann J, Zeiher AM, Dimmeler S (2004). Antioxidant effects of statins via S-nitrosylation and activation of thioredoxin in endothelial cells: a novel vasculoprotective function of statins. Circulation.

[b18-ehp-119-481] Kinney PL, Aggarwal M, Northridge ME, Janssen NA, Shepard P (2000). Airborne concentrations of PM_2.5_ and diesel exhaust particles on Harlem sidewalks: a community-based pilot study. Environ Health Perspect.

[b19-ehp-119-481] Liang YJ, Shyu KG, Wang BW, Lai LP (2008). Simvastatin inhibits C-reactive protein-induced pro-inflammatory changes in endothelial cells by decreasing mevalonate pathway products. Cardiology.

[b20-ehp-119-481] Lim SC, Caballero AE, Smakowski P, LoGerfo FW, Horton ES, Veves A (1999). Soluble intercellular adhesion molecule, vascular cell adhesion molecule, and impaired microvascular reactivity are early markers of vasculopathy in type 2 diabetic individuals without microalbuminuria. Diabetes Care.

[b21-ehp-119-481] Madrigano J, Baccarelli A, Wright R, Suh H, Sparrow D, Vokonas P (2010). Air pollution, obesity, genes, and cellular adhesion molecules. Occup Environ Med.

[b22-ehp-119-481] Maynard D, Coull BA, Gryparis A, Schwartz J (2007). Mortality risk associated with short-term exposure to traffic particles and sulfates. Environ Health Perspect.

[b23-ehp-119-481] Montecucco F, Burger F, Pelli G, Poku NK, Berlier C, Steffens S (2009). Statins inhibit C-reactive protein-induced chemokine secretion, ICAM-1 upregulation and chemotaxis in adherent human monocytes. Rheumatology (Oxford).

[b24-ehp-119-481] O’Neill MS, Veves A, Sarnat JA, Zanobetti A, Gold DR, Economides PA (2007). Air pollution and inflammation in type 2 diabetes: a mechanism for susceptibility. Occup Environ Med.

[b25-ehp-119-481] O’Neill MS, Veves A, Zanobetti A, Sarnat JA, Gold DR, Economides PA (2005). Diabetes enhances vulnerability to particulate air pollution-associated impairment in vascular reactivity and endothelial function. Circulation.

[b26-ehp-119-481] Park SK, O’Neill MS, Vokonas PS, Sparrow D, Spiro A, Tucker KL (2008). Traffic-related particles are associated with elevated homocysteine: the VA Normative Aging Study. Am J Resp Crit Care Med.

[b27-ehp-119-481] Pradhan AD, Rifai N, Ridker PM (2002). Soluble intercellular adhesion molecule-1, soluble vascular adhesion molecule-1, and the development of symptomatic peripheral arterial disease in men. Circulation.

[b28-ehp-119-481] Rana JS, Arsenault BJ, Després JP, Côté M, Talmud PJ, Ninio E (2011). Inflammatory biomarkers, physical activity, waist circumference, and risk of future coronary heart disease in healthy men and women. Eur Heart J.

[b29-ehp-119-481] Ren C, Park SK, Vokonas PS, Sparrow D, Wilker E, Baccarelli A (2010). Air pollution and homocysteine: more evidence that oxidative stress-related genes modify effects of particulate air pollution. Epidemiology.

[b30-ehp-119-481] Ridker PM, Buring JE, Cook NR, Rifai N (2003). C-reactive protein, the metabolic syndrome, and risk of incident cardiovascular events: an 8-year follow-up of 14719 initially healthy American women. Circulation.

[b31-ehp-119-481] Salvi S, Blomberg A, Rudell B, Kelly F, Sandstrom T, Holgate ST (1999). Acute inflammatory responses in the airways and peripheral blood after short-term exposure to diesel exhaust in healthy human volunteers. Am J Resp Crit Care Med.

[b32-ehp-119-481] Schneider A, Neas L, Herbst MC, Case M, Williams RW, Cascio W (2008). Endothelial dysfunction: associations with exposure to ambient fine particles in diabetic individuals. Environ Health Perspect.

[b33-ehp-119-481] Schwartz J (2001). Air pollution and blood markers of cardiovascular risk. Environ Health Perspect.

[b34-ehp-119-481] Schwartz J, Park SK, O’Neill MS, Vokonas PS, Sparrow D, Weiss S (2005). Glutathione-*S*-transferase M1, obesity, statins, and autonomic effects of particles: gene-by-drug-by-environment interaction. Am J Respir Crit Care Med.

[b35-ehp-119-481] U.S. Census Bureau, Population Division (2005). Interim Projections of the Population by Selected Age Groups for the United States and States: April 1, 2000 to July 1, 2030.

[b36-ehp-119-481] Yorifuji T, Kashima S, Tsuda T, Takao S, Suzuki E, Doi H (2010). Long-term exposure to traffic-related air pollution and mortality in Shizuoka, Japan. Occup Environ Med.

[b37-ehp-119-481] Zanobetti A, Schwartz J (2002). Cardiovascular damage by airborne particles: are diabetics more susceptible?. Epidemiology.

[b38-ehp-119-481] Zeka A, Sullivan JR, Vokonas PS, Sparrow D, Schwartz J (2006). Inflammatory markers and particulate air pollution: characterizing the pathway to disease. Int J Epidemiol.

